# A Rare Case of a High-Grade Appendiceal Mucocele Causing Colocolonic Intussusception

**DOI:** 10.7759/cureus.96117

**Published:** 2025-11-05

**Authors:** Vinita R Chaudhari, Indrani Mandal, Nazli M Ullah, Suresh B Pillai

**Affiliations:** 1 General Surgery, United Lincolnshire Hospitals Trust, Lincoln, GBR; 2 Surgery, Queens Medical Centre, Nottingham, GBR; 3 Colorectal Surgery, United Lincolnshire Hospitals Trust, Lincoln, GBR, Lincoln, GBR

**Keywords:** appendiceal mucocele, colorectal intussusception, emergency colorectal surgery, high grade appendiceal mucinous neoplasm, incidental appendiceal mucocele

## Abstract

Appendiceal mucoceles are rare, accounting for less than 0.3% of all appendiceal pathology. High-grade appendiceal mucinous neoplasms (HAMNs) are a specific subtype with a significant risk for malignancy and poor prognosis, representing approximately 20% of appendiceal mucinous tumors. Complications of an appendiceal mucocele include rupture, malignancy, intestinal obstruction, and, rarely, colonic intussusception. Here, we present the case of a 31-year-old female with an asymptomatic appendiceal mucocele leading to intussusception. Following a computed tomography abdominal pelvis (CTAP) scan confirming the diagnosis, she underwent an emergency right hemicolectomy. Histopathology revealed a high-grade mucinous neoplasm. This report highlights the diagnostic challenges, surgical management, and histopathological findings of this rare entity.

## Introduction

Appendiceal mucoceles are uncommon pathological findings characterised by the gradual build-up of mucin within the appendiceal lumen, leading to progressive distension of the organ [1]. The causes of this condition vary and may include both non-neoplastic processes, such as simple retention cysts, and neoplastic changes, including mucinous cystadenomas or cystadenocarcinomas. Adult intussusception, meanwhile, is a rare clinical event that accounts for only 1-5% of intestinal obstructions and is most often linked to an underlying lesion acting as a pathological lead point, frequently of neoplastic origin [2].

While many appendiceal mucoceles are benign, a proportion can undergo neoplastic transformation, giving rise to malignant forms such as high-grade appendiceal mucinous neoplasms (HAMNs) [3]. These neoplasms represent roughly 10-15% of appendiceal tumours and are known for their more aggressive course. Their clinical importance stems from their potential for local tissue invasion and spread within the peritoneal cavity, which may culminate in pseudomyxoma peritonei--a condition associated with significant morbidity [4].

Although rare, cases in which an appendiceal mucinous tumour leads to intussusception can be particularly challenging to diagnose and manage. The symptoms are often vague and easily mistaken for more common abdominal conditions, which can delay diagnosis. For this reason, maintaining a high index of suspicion and employing careful imaging evaluation are crucial for early detection. Surgical management remains the mainstay of treatment, providing both symptom relief and a means for definitive histopathological diagnosis, while also helping to prevent potential peritoneal dissemination [5].

## Case presentation

**Figure 1:**
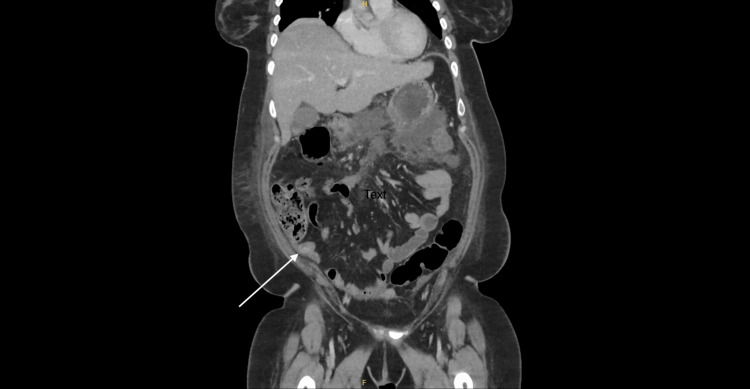
Incidental finding: mucocele of the appendix (coronal view)

**Figure 2:**
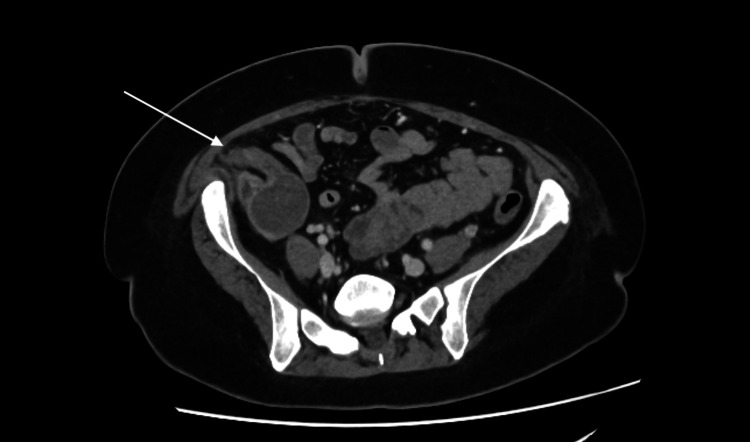
Incidental finding: mucocele of the appendix (axial view)

A 31-year-old female initially presented with acute cholecystitis and choledocholithiasis. Imaging via CT and magnetic resonance cholangiopancreatography (MRCP) revealed an incidental appendiceal mucocele (Figure 1, Figure 2). She was referred to the colorectal multidisciplinary team (MDT) for elective surgery, scheduled for April 2024.

In the interim, she experienced post-procedural complications, including post-ERCP pancreatitis and insulin-dependent diabetes mellitus. Despite conservative management, follow-up computed tomography abdominal pelvis (CTAP) demonstrated persistence of the appendiceal mucocele.

**Figure 3:**
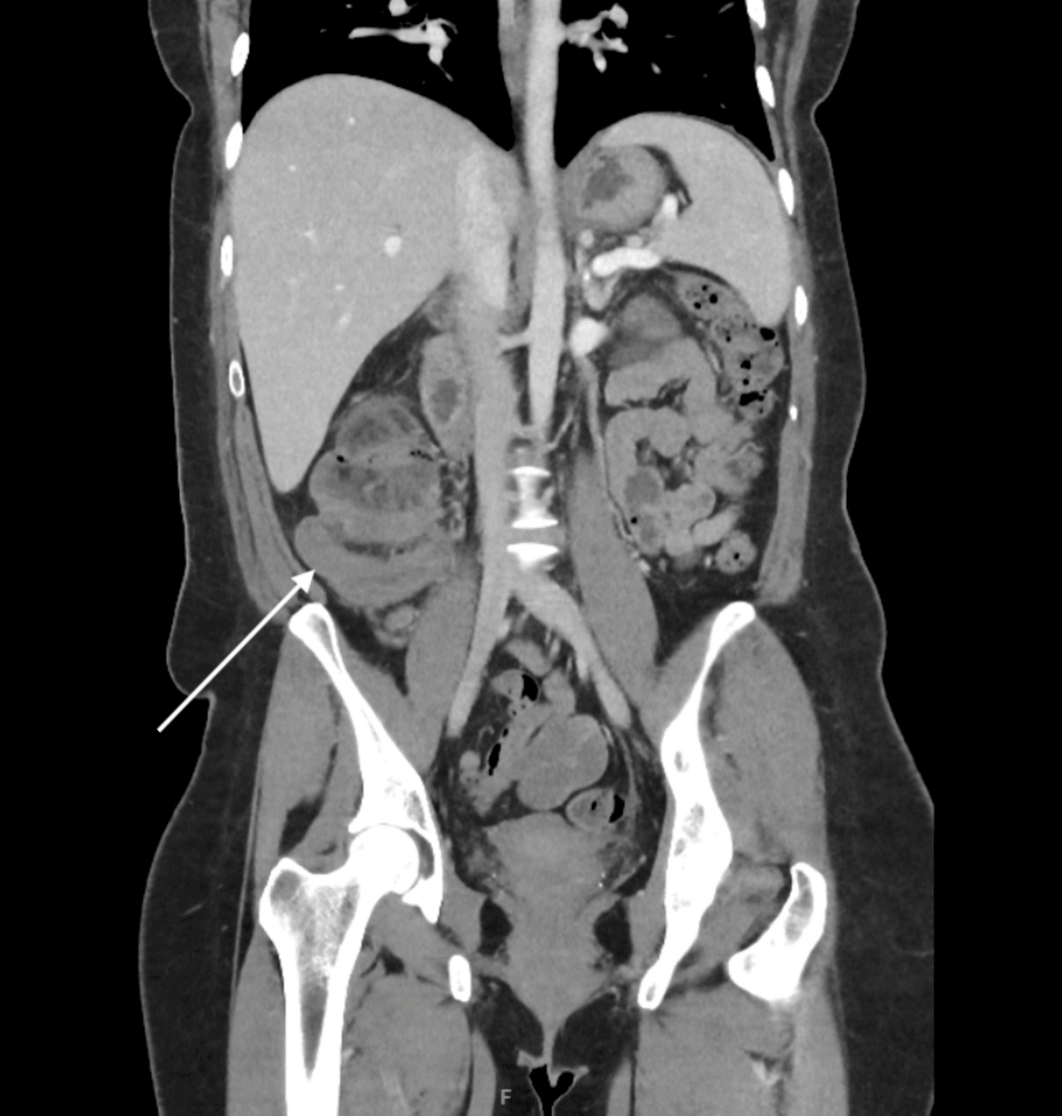
Colocolonic intussusception with the appendiceal mucocele as the leading point

On 6 March 2024, the patient developed acute right-sided abdominal pain. CTAP identified colonic intussusception, a colocolonic fistula, and the previously noted appendiceal mucocele acting as the lead point (Figure 3). The imaging raised suspicion of an early mucinous appendiceal malignancy, with a few enlarged mesenteric lymph nodes. Emergency laparotomy revealed omental adhesions, intussusception extending from the hepatic flexure to the caecum, and a distended, thickened appendix. Multiple enlarged lymph nodes were noted. Surgical intervention comprised adhesion release, intussusception reduction, right colon mobilisation, duodenal preservation, and right hemicolectomy [1,2,5]. Resection margins were clear, with no evidence of metastatic disease.

Pathological findings

**Figure 4:**
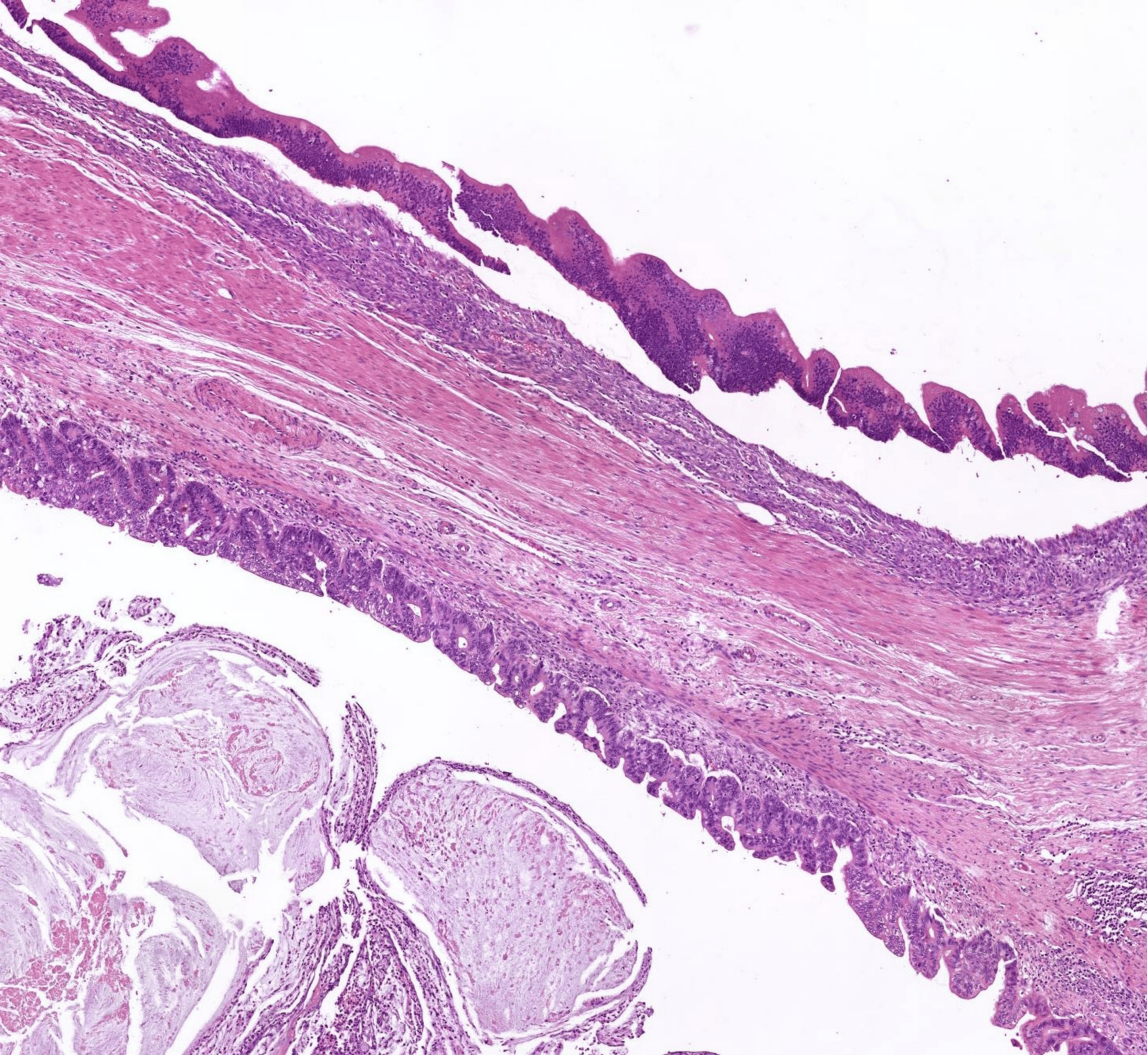
Cells lining the appendix with low-grade nuclear atypia (top) and high-grade nuclear atypia (bottom)

**Figure 5:**
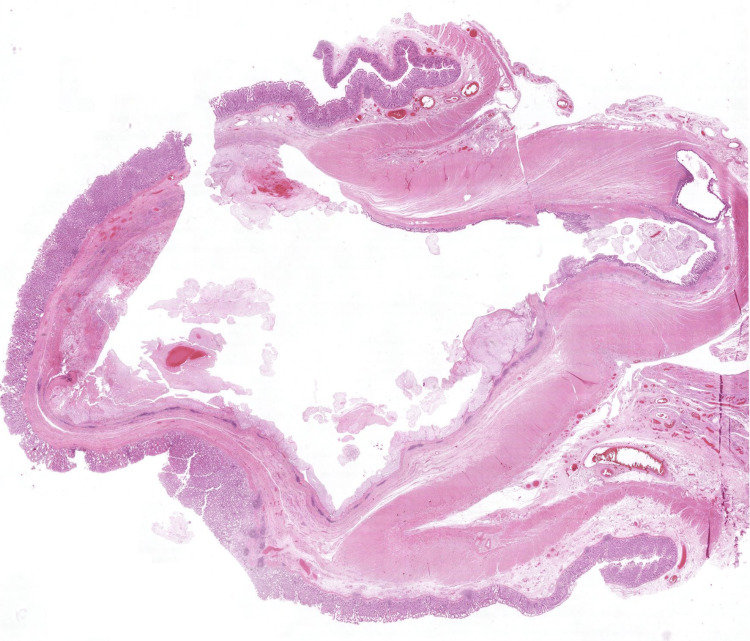
Caecum with dilated appendix containing luminal mucin

Histopathological examination demonstrated a markedly dilated appendiceal lumen containing mucin. The mucosa exhibited tufted mucinous epithelium and areas of flattening with lamina propria loss. Low-grade and high-grade nuclear atypia were evident (Figure 4). Approximately 20% of the tumour displayed high-grade features, including cribriform architecture, increased mitotic activity, nuclear stratification, and pronounced cytological atypia. Tumour rupture with mucin dissection through the appendiceal wall and presence on the serosal surface was observed [4]. There was additional rupture through the caecal wall, exhibiting a pushing invasive front (Figure 5). The final diagnosis was a HAMN, pT4a, grade 2, with longitudinal and non-peritonealised margins exceeding 30 mm. All 15 lymph nodes were negative for metastasis [4,5].

## Discussion

Although uncommon, appendiceal mucoceles present considerable diagnostic and therapeutic challenges [1,3]. HAMNs are particularly concerning due to their malignant potential and unfavourable prognosis [4]. The presentation of a mucocele causing intussusception in adults is exceedingly rare and warrants urgent surgical intervention [1,3]. CT imaging remains pivotal in the preoperative diagnosis, with features such as target or sausage-shaped lesions, hypodense luminal content, and occasional calcifications being indicative [1,3].

Surgical resection is the definitive management, particularly in adult intussusception secondary to a neoplastic lead point [1,2,5]. The extent of resection should be guided by tumour size, involvement of the appendiceal base, and suspicion of malignancy [2,5]. Histopathological assessment is essential to confirm the diagnosis and determine tumour grade, guiding subsequent treatment and follow-up strategies [4].

Prognosis varies, with recurrence risk heightened in the presence of rupture or mucin spillage [3,5]. Tumour size, base involvement, lymph node status, and margin clearance are critical prognostic indicators [5]. Prompt detection and complete resection remain paramount in optimising patient outcomes.

In contrast to management options, when an appendiceal intussusception does not accompany the possibility of other malignancies, colonoscopic reduction followed by an operation could better improve the inflammation than laparotomy only. This strategy is also deemed to minimise the extent of operation of the leading point, thereby enabling the patient to recover more rapidly [6].

## Conclusions

This report presents an exceptionally rare instance of a high-grade appendiceal mucocele causing colocolonic intussusception, emphasising the need for vigilance when evaluating atypical abdominal presentations. The case illustrates how subtle radiological clues, interpreted with sound clinical judgement, can unveil a potentially malignant process before catastrophic complications occur. Early surgical management, supported by precise histopathological assessment, proved decisive in achieving a favourable outcome. Such cases reinforce the importance of multidisciplinary collaboration and remind clinicians that even the rarest pathologies merit timely recognition and meticulous care.
